# Confounding fuels misinterpretation in human genetics

**DOI:** 10.1098/rspb.2025.1615

**Published:** 2025-11-05

**Authors:** John W. Benning, Jedidiah Carlson, Olivia S. Smith, Ruth G. Shaw, Arbel Harpak

**Affiliations:** ^1^ Department of Ecology and Evolutionary Biology, Cornell University, Ithaca, NY, USA; ^2^ Department of Botany, University of Wyoming, Laramie, WY, USA; ^3^ Department of Integrative Biology, The University of Texas at Austin, Austin, TX, USA; ^4^ Department of Population Health, The University of Texas at Austin, Austin, TX, USA; ^5^ Department of Mathematics, Statistics, and Computer Science, Macalester College, Saint Paul, MN, USA; ^6^ Department of Ecology, Evolution and Behavior, University of Minnesota, Minneapolis, MN, USA

**Keywords:** confounding, human, genetics, social science genomics, association studies, genomic prediction

## Abstract

The scientific literature has seen a resurgence of interest in genetic influences on human behaviour and socioeconomic outcomes. Such studies face the central difficulty of distinguishing possible causal influences, in particular genetic and non-genetic ones. When confounding between possible influences is not rigorously addressed, it invites over- and misinterpretation of data. We illustrate the breadth of this problem through a discussion of the literature and a reanalysis of two examples. The first paper we discuss suggested that patterns of similarity in social status between relatives indicate that social status is largely determined by one’s DNA. Our reanalysis shows that the paper’s conclusions are based on the conflation of genetic and non-genetic transmission (for example, of wealth) within families. The second paper we discuss posited that genetic variants underlying bisexual behaviour are maintained in the population because they also affect risk-taking behaviour and thereby confer an evolutionary fitness advantage through increased sexual promiscuity. In this case, too, our reanalysis shows that, though possible explanations cannot be distinguished, only one is chosen and presented as a conclusion. We discuss how issues of confounding apply more broadly to studies that claim to establish genetic underpinnings to human behaviour and societal outcomes.

## 1. Introduction

**Figure 1.**
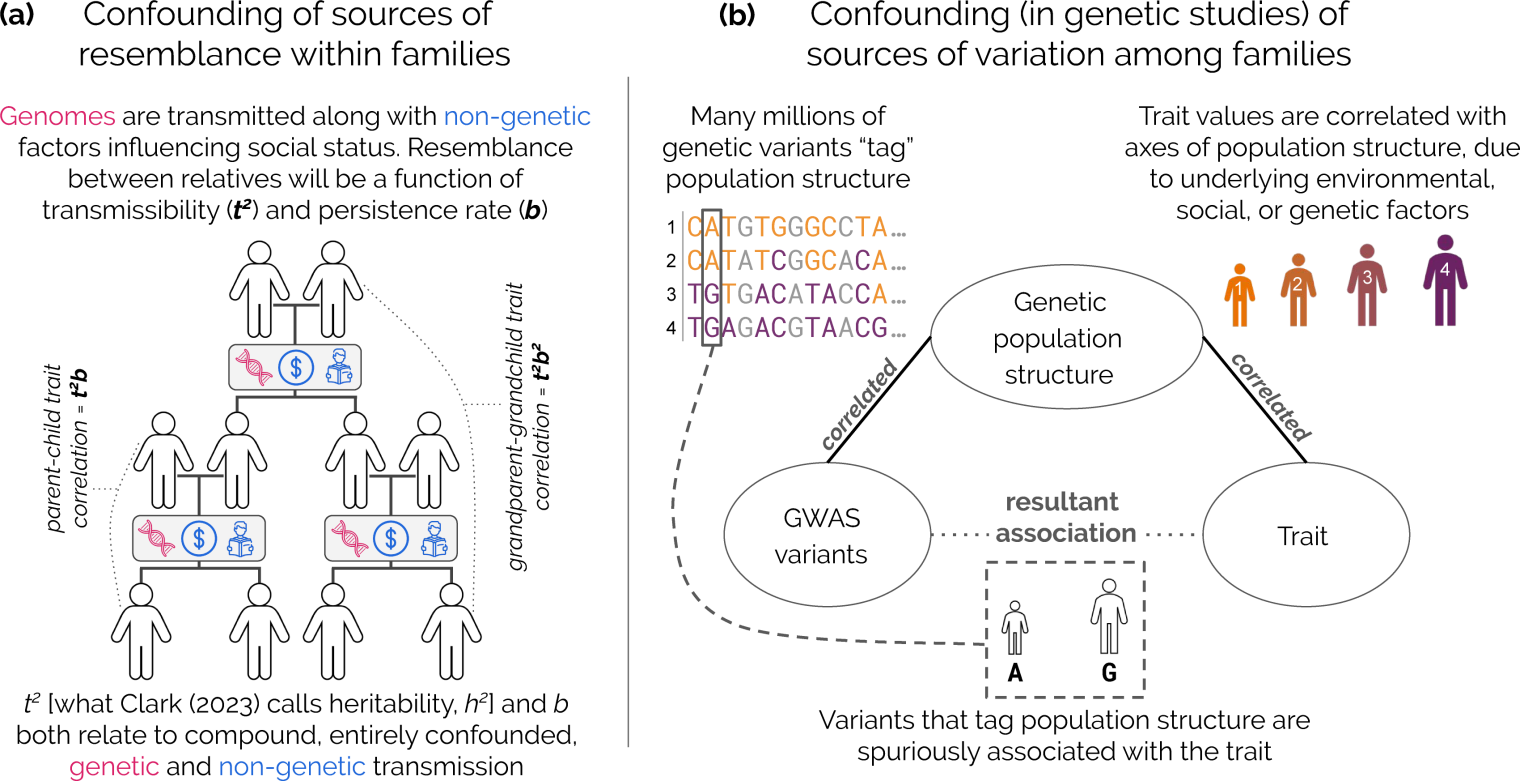
Confounding between genetic and non-genetic factors influencing traits. (a) Confounding within families. Non-genetic transmission can parallel genetic transmission and their respective effects are confounded in observational data. Illustrated is a model where a trait value is the sum of an inherited component from parents and random noise. Under this model, the expected resemblance between relatives depends on transmissibility (*t^2^*, the portion of trait variation attributable to the transmitted component) and a rate of decay across genealogical distance (the ‘persistence rate,’ *b,* which increases with increasing degree of assortative mating). Ignoring the confounding of genetic and non-genetic transmission in the data, Clark [3] misassigns all transmission as genetic heritability and all assortative mating to be on a latent ‘social genotype’. (b) Confounding among families induces biases in genome wide association studies (GWAS). ‘Population structure confounding’ in genomic data relates to correlations between the structure of genetic relatedness in a GWAS sample (exemplified by the orange-to-purple gradient) and the phenotype studied. Here we show genetic sequences from individuals 1−4 at top left, with their attendant phenotypes (height) at top right. For a given genetic variant, individuals with purple alleles will tend to be taller than those with orange alleles, regardless of the variant’s causal effect on height. This confounding affects any variants that reflect this axis of genetic population structure—typically many millions of variants. While researchers often use methods that adjust for population structure in an attempt to avoid spurious associations, the extent of residual confounding in GWAS remains unclear.

People vary remarkably in behaviour and social outcomes. This variation sparks curiosity about its causes, and for the past 150 years, scholars have debated the extent to which it arises owing to underlying genetic differences. In the nineteenth century, Galton [1] found strong resemblance between parents and their offspring in measures of social status and, on that basis, inferred that genetics is the most likely root cause, a school of thought described broadly as ‘hereditarianism’ [2]. As is now well appreciated, Galton’s inference neglected the fact that parents transmit not only genetic material to their offspring, but also wealth, place of residence, knowledge, religion, culture and more. For such attributes, transmission within families can parallel genetic transmission (figure 1a; [3–21]), often leading genetic and non-genetic transmission to be indistinguishable in observational data. A long history of scholarship has highlighted this type of confounding and how it impedes inference of the causes of phenotypic variation [22–30]. Studies without molecular genetic data are particularly susceptible to confounding, because they offer little to no signal that could be argued to reflect only genetic or only non-genetic transmission [22,23,26,28,31].

This manuscript is motivated by the fact that confounding is still frequently overlooked or downplayed in reports about genetic causes of human behaviour and socioeconomic outcomes (box 1; [32–37]). Through reanalysis of two datasets, we demonstrate instances of confounding that underlie misinterpretation in current, high-profile papers that have been drawing media attention (ranking in the 99th percentile of Altmetric Attention Scores for papers of a similar age). We begin with a recent publication that made claims about genetic determinism of social status [3] in the absence of molecular genetic data or viable strategies for disentangling genetic from non-genetic contributions (box 1i). We then discuss sources of confounding undermining causal inference based on genome wide association studies (GWAS) for behaviour and social outcomes, with a focus on confounding via population stratification (box 1ii,iii). Lastly, we consider the impacts of related errors in causal inference stemming from data preparation and other analysis choices (‘analytical confounding’; box 1iv). We illustrate these problems using a recent study [32] that purported to explain the evolutionary maintenance of genetic variation affecting bisexual behaviour.

## 2. Confounding fuels hereditarian fallacies

A recent publication [3] analysed familial correlations in a dataset of socioeconomic measures (e.g. occupational status, house value, literacy) from a selection of records spanning the eighteenth to twenty-first centuries in England. In it, a quantitative genetic model is fitted to these observed correlations ([38,39]; electronic supplementary material, note S1). Based on this fit, [3] infers that social status persists intergenerationally because of strong assortative mating on a status-determining genotype (or ‘social genotype’ as the author has termed it in previous work [40]). Further, the paper argues that the persistence of social status within families—and persistence of differences in status among families—have been largely unaffected by changes in social policy in the last four centuries. In a subsequent commentary about this work [41], the author presents the results of [3] as providing strong support for a hereditarian interpretation. In doing so, he appeals to the metaphor of a ‘genetic lottery’ underlying social outcomes.

**Figure 2.**
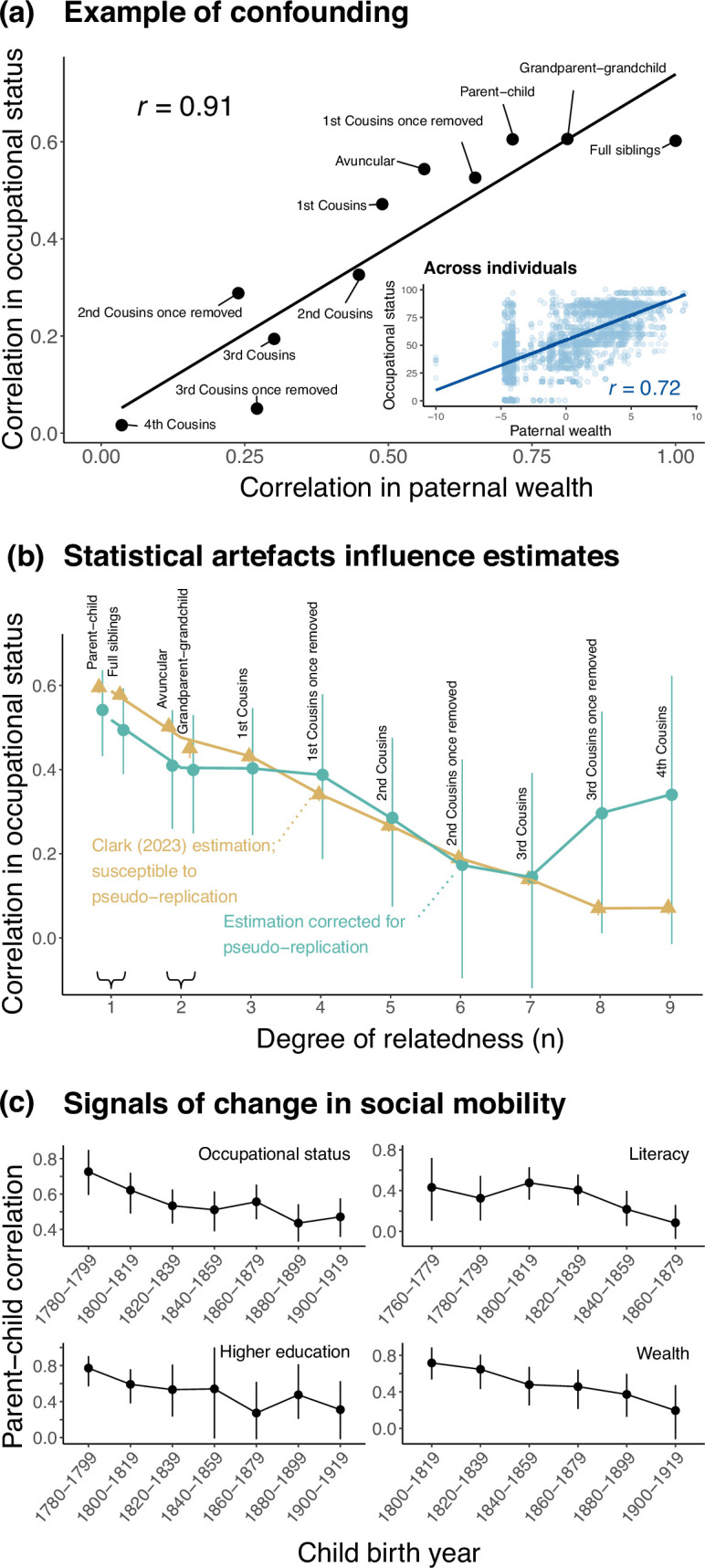
Reanalyses of data from Clark [3] challenge the paper’s claims. (a) Example of confounding between genetic and non-genetic transmission. The relationship between paternal wealth and a measure of social status suggest at least one source of confounding between genetic and non-genetic transmission. Across relative pairs, correlation in occupational status is highly correlated (Pearson’s *r* = 0.91) with those relatives’ correlation in paternal wealth. Inset shows that individual occupational status is strongly correlated (Pearson’s *r* = 0.72) with father’s wealth. Excluding outliers in paternal wealth (1.5× interquartile range rule) had only a small impact (a change of less than 0.01) on the Pearson correlations reported here. Plots show data for individuals born 1780−1859 and their fathers. For the cohort of individuals born after 1859, the correlations are *r* = 0.72 for relative pairs and *r* = 0.60 for individuals. Clark [3] estimated wealth from probate records. Log wealth at death was mean-centred by subtracting the mean log wealth for each decade. Individuals not probated owing to insufficient wealth were assigned a value of half the minimum probate requirement for the time period. (b) Pseudoreplication distorted estimates of familial correlations. Familial correlations (95% confidence interval (CI)) in occupational status (1780−1859) using the approach employed by [3] (in gold) involved pervasive, non-uniform pseudoreplication (electronic supplementary material, note S6). For example, the (1780−1859) occupational status correlation for fourth cousins is calculated from 17 382 pairs, derived from only 1878 unique individuals. In teal we show conservative estimates using only a single relative pair per surname (means and 95% CI over 1000 bootstrap samples are plotted for each familial correlation), which are therefore not susceptible to pseudoreplication. Distant cousins show dramatically higher correlations after adjusting for pseudoreplication. We note that additional biases may exist that are not addressed with this adjustment (electronic supplementary material, note S6). (c) Signals of change in social mobility. Parent-offspring correlations in multiple status measures generally decrease over time in Clark’s [3] data, in contrast to claims of stagnant social mobility made in the original paper. To mitigate pseudoreplication, we calculated correlations using one pair from each surname (as in (b)). Shown are average correlations (95% CI) across 500 bootstrap iterations of correlation estimation. The electronic supplementary material, figure S13 shows two complementary analyses estimating correlations either without accounting for pseudoreplication, or using percentile ranks—both result in similar trends.

Here, we discuss the failure to account for the confounding of genetic and non-genetic transmission (figure 1a) that, together with other core flaws of the analysis (figure 2a,b), fuels the hereditarian claims in [3] (see our discussion of other misinterpretations, errors and incongruencies in [3] in the electronic supplementary material, notes S2−S7, tables S1−S3, and figures S1−S13). We also demonstrate that familial status correlations varied substantially over the time period examined, generally decreasing (figure 2c). This finding contrasts with conclusions in [3], based on the same data, that social mobility has been stagnant. As we show below, the analyses in [3] do not establish the contribution of genetics to social status.

**Figure:**
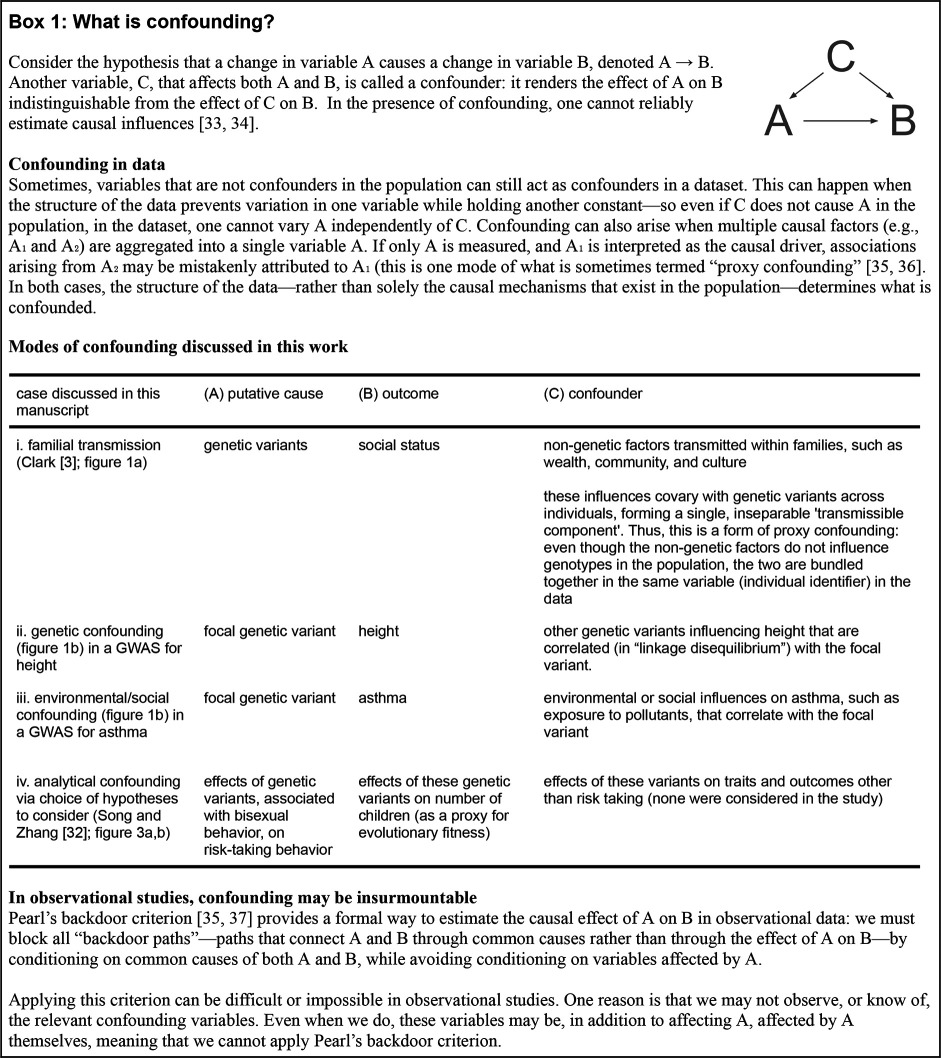


### (a) Confounding between genetic and non-genetic transmission

Inferences in [3] are based on a linear regression model derived from quantitative-genetic theory developed by R.A. Fisher ([38,39]; electronic supplementary material, note S1) and the model

(2.1) P=G+E,

where an individual’s phenotype, P, is the sum of separable genotypic (G) and environmental (E) influences on it. Because genes are transmitted from parents to offspring, genetic parameters can be inferred from correlations between relatives, on the condition that environmental influences are random and independent of genotypes. Fisher [38] formally showed that under this model, the expected correlation in a trait between pairs of individuals of a defined relationship is a function of the genealogical relationship between the relatives, the trait’s heritability ($h^{2}$), and the extent of assortative mating in the population (b). ($h^{2}$ is the fraction of phenotypic variance due to additive genetic variance, commonly referred to as ‘narrow-sense’ heritability.)

Crucially, to interpret the model parameters $h^{2}$ and b as relating to genetic effects, Fisher’s model assumes that there are no non-genetic (material, environmental or cultural) influences on a trait that are systematically shared or transmitted between relatives. This assumption is valid in an experimental setting, for instance, in which genotypes are randomized with regard to environment. However, in humans that assumption is nonsensical. Non-genetic transmission is ubiquitous for social and behavioural traits. Traits may be transmitted directly between relatives (e.g. literate parents teaching their children how to read; [6]), or via indirect mechanisms such as ‘ecological inheritance,’ where the trait value of an offspring is influenced by the environmental conditions bestowed by their parents (e.g. familial wealth influencing educational opportunities; [9,42]). When genotypes cannot be randomized over environments, true genetic effects are much more difficult to separate from other factors underlying phenotypic resemblance between relatives [23]. In [3], for instance, the assumption of no systematic non-genetic transmission implies that similarity in house value among relatives (one of the measures of social status analysed) is solely owing to shared genes, and does not arise from similarity in parental wealth, the inheritance of wealth or property, or having learnt from one’s relatives about investment.

In fact, we found evidence of strong confounding between genetic and non-genetic contributions to familial resemblance in the data used in [3]. The paper acknowledges the inheritance of material wealth from one’s parents as an example of non-genetic transmission only when treating wealth itself as the focal status measure. For other measures studied, the effect of familial wealth on social status is ignored. However, familial wealth can obviously influence a wide range of conditions that affect offspring (e.g. healthcare, place of residence, access to tutors, social circles, etc.; [43–47]). Consistent with this intuition, we found that all seven status measures analysed in [3] are substantially correlated with paternal wealth (Pearson’s *r* ranging from 0.19 to 0.66; mean *r* = 0.36; all $p<2\;\times 10^{-16}$; electronic supplementary material, table S2; figure 2a). Closer relatives tend to have more similar paternal wealth, and the similarity in paternal wealth between relatives predicts their similarity in occupational status extremely well (Pearson’s *r* = 0.91; figure 2a). Thus, the effect of transmission of genes and that of parental wealth on familial similarity in social status are confounded in these data. Apart from wealth, numerous other non-genetic factors may contribute to familial correlations [48,49]. Two post hoc analyses are presented in [3] in an attempt to rule out non-genetic contributors to familial resemblance in social status. In the electronic supplementary material, note S4, we detail why these analyses are uninformative as to the strength of non-genetic effects on resemblance in social status between relatives (electronic supplementary material, figure S1).

This confounding invalidates the interpretation of the model parameters offered in [3] as pointing to identifiable genetic contributions (electronic supplementary material, note S1). In particular, in the presence of such confounding, the interpretation of *G* and *E* in equation (2.1) as transmissible genetic (*heritable*) and random non-genetic effects on a phenotype, respectively, no longer holds. Instead, they can be interpreted as a *transmissible* component and a random, non-transmissible component. Consequently, the parameter interpreted in [3] as narrow-sense heritability, $h^{2}$, is in fact an estimate of the ‘total transmissibility’ of a trait, $t^{2}$, the proportion of trait variance attributable to an unknown compound of transmissible influences on the traits, including genes, culture, wealth, environment, etc. [11,13]. The second key parameter, m, which [3] interpreted as the ‘spousal correlation in the underlying genetics,’ does not represent a genetic correlation between mates. It is instead the spousal correlation in the transmissible component of the trait. m is derived from the ‘intergenerational persistence rate,’ $b=\frac{1+m}{2}$, estimated from the regression model. The expected correlation for a given kinship pair is equal to $t^{2}b^{n}$, where n denotes genealogical distance (figure 1a). Note that the parameterization of b for father–son and grandparent–grandchild relationships also depends on the degree of assortative mating with respect to the focal trait itself; see the electronic supplementary material, note S1. The conflation of genetic and non-genetic transmission helps to explain why the model parameters estimated in [3], which are claimed to represent quantitative genetic parameters $h^{2}$ and m, are much higher than estimates of these same parameters from studies that strive to account for confounding (e.g. [20,50,51]).

Conclusions in [3] about the insensitivity of social standing to policy and sociopolitical context rest on the similarity of estimates of the parameter b across status measures and across time. The claim [3] that this stability is owing to strong assortative mating on a genetic factor for ‘social ability’ does not hold, given that both genetic and non-genetic factors are transmitted within families. The estimate of m tells us nothing about genetic versus non-genetic contributions to assortment, and b tells us nothing about the cause of within-family persistence of social status (figure 1a; electronic supplementary material, note S1).

Regardless of whether owing to genetic causes or not, a striking claim in [3, pg 1] is that *‘The vast social changes in England since the Industrial Revolution, including mass public schooling, have not increased, in any way, underlying rates of social mobility*’. In point of fact, we found that the estimates of familial correlations in [3], and, in turn, estimates of the persistence rate, are heavily affected by statistical artefacts (figure 2b; electronic supplementary material, note S6). Furthermore, we show that across status measures, parent–offspring correlations—an established measure of social mobility [52,53]—generally decrease over time (figure 2c; electronic supplementary material, note S7). How could the new measure, ‘persistence rate’, used by [3] lead to such contrasting conclusions? Clark [3] offers neither justification for why this measure reflects social mobility, nor explanation for the discrepancies with established measures of mobility used in other literature (e.g. [54]) and applied to the same data.

Some readers have already taken arguments in [3] as compelling evidence that social status is largely caused by genetic factors [55–59]. Yet the assumptions and interpretations in [3] ignore a century of quantitative-genetic theory, previous empirical evidence for confounding, and the fallacies that arise when confounding is ignored [14,18,22,23,27,49,60–67], as well as patterns in the paper’s own data that conflict with the interpretations presented. In this regard, we emphasize that [3] does not merely overstate the findings: the model parameters are misconstrued and the pervasive confounding of genetic and non-genetic transmission is not addressed.

## 3. Are modern genomic studies less susceptible to confounding?

In relying solely on observational phenotypic data and assuming that transmission in families is solely genetic, [3] is similar in spirit to studies carried out by Francis Galton a century and a half ago. One might hope that the inferential flaws described above are remedied in studies that use large genomic datasets and employ state-of-the-art statistical methods to adjust for confounding. As we outline, however, the same concerns remain broadly applicable; confounding is still poorly understood and often underplayed in the literature.

### (a) Confounding in genomic studies is poorly understood

Human geneticists have long appreciated that there are myriad ways by which a genetic variant may be associated with a trait or outcome [27,68,69]. A key example is ‘population stratification’ in genomic data (e.g. in GWAS) wherein patterns of genetic similarity in a sample are correlated with the phenotype studied (figure 1b). Possible reasons for this correlation include social, environmental (box 1iii) or genetic (box 1ii) factors, contemporary and historical. Typically, the specific causes are unknown. These same axes of genetic similarity (‘population structure’) are reflected in the frequencies of numerous genetic markers that may be tested for association with a trait in a GWAS. Consequently, any such markers will tend to be correlated with the trait, even if only a subset (or in fact none) of the variants causally affect it (figure 1b).

Consider, for example, a GWAS conducted to identify genetic risk factors for asthma in a sample of people from the United States (US) of either primarily European American genetic ancestry or primarily African American genetic ancestry. There are many millions of variants in the genome that significantly differ in frequency between these groups. At the same time, African Americans in the US are systematically exposed to higher levels of air pollution [70], an environmental risk factor for asthma. If confounding is not adequately addressed, the GWAS would lead us to conclude erroneously that ‘African American genetics’ predispose one for asthma (box 1iii). Regardless of what drives population stratification, it can result in biased estimates of the individual effects of numerous genetic markers that tag these axes of population structure (figure 1b).

Human geneticists use various methods to adjust for confounded associations. However, confounding may persist, despite application of these methods (‘residual confounding’). In 2019, we and other researchers discovered that genetic effect estimates in the largest GWASs for height—the most extensively studied polygenic trait—were biased owing to residual confounding [65,66]. It is plausible that this confounding is, at least in part, ‘genetic confounding’: the effect estimated for each individual variant was biased by the effects of many other variants (box 1iii). Regardless of the source of confounding, it became clear that while the bias for each individual genetic variant was slight, it was systematic across variants. Consequently, when researchers summed over signals from many genetic variants, they also summed over systematic biases. This led to erroneous conclusions in many studies (as detailed in [18,65,66]). Further research has suggested that residual confounding may affect many GWASs, in particular for social outcomes and traits that are heavily influenced by social context [27,67,69,71–73].

Even so, it is often impossible to explicitly demonstrate a lack of statistical identifiability or to pinpoint the specific confounders in a study. To our knowledge, no general method exists for the detection and quantification of confounding in GWAS data. In the absence of an omnibus litmus test for confounding, the answer cannot be to reduce the burden of proof for causal narratives.

### (b) Confounding in genomic studies is downplayed

Papers often imply that confounding is completely remedied by current methods, despite evidence to the contrary [65–67,69,71,73–83]. Some methods to estimate genetic parameters have grown in popularity even after they were shown to be susceptible to confounding, with this susceptibility rarely mentioned as a caveat (see discussions in [65,72,73,77,84,85]). In other cases, confounding is acknowledged as a potential limitation, but its impact on the reported results (and their interpretation) is downplayed or obscured (see for instance, [86,87]).

As one example, consider the reporting of evidence for genetic effects from standard GWASs versus family-based studies. Family studies identify genotype-trait associations within, instead of among, families. This approach greatly mitigates many sources of confounding [67,69,88]. Family studies have yielded estimates of genetic effects on behaviour or social outcomes that are substantially weaker than those estimated from standard GWASs [50,67,71,73,89–92]. Reporting practices tend to downplay this point by using evidence from family studies as support for *existence* of a causal genetic effect, while continuing to rely on the magnitude of effects estimated using standard GWAS [62]. Such reporting choices mislead by presenting signals susceptible to confounding as measures of genetic causality.

### (c) Confounding in complex traits genetics: aggregation of many small biases

Quantitative geneticists acknowledge residual confounding as an unsolved problem. However, in practice, researchers face incentives to publish their inferences of genetic associations that are vulnerable to confounding. With polygenic (or ‘complex’) traits, the usual focus of these studies, the genetic contribution to trait variation is in large part composed of numerous genetic variants with individually small effects. Researchers often wish to leverage weaker and weaker genetic associations to capture these highly polygenic signals. At the same time, confounding tends to be aggravated as more weakly associated variants are considered [67,81]. Thus, in the pursuit of understanding polygenic effects, researchers may face a trade-off between explaining a smaller part of the phenomenon under study in a causally rigorous way, versus accounting for a seemingly larger part, at the price of unknown biases introduced by confounding.

An example of this trade-off lies in genetic trait prediction with so-called ‘polygenic scores’ [93]. Polygenic scores based on more variants, including weakly-associated ones, may be preferred by researchers because they often attain higher prediction accuracy than polygenic scores that are limited to confident associations. However, polygenic scores that include many weakly-associated variants are plausibly more susceptible to underappreciated axes of confounding [67,81,94]. Subsequent ‘consumers’, including clinicians, researchers, policymakers and the general public, may then assume these polygenic scores capture strictly direct genetic effects; the possibility of confounding is rarely acknowledged. Consider, for instance, a hypothetical pre-implantation genetic test using a polygenic score based on the asthma GWAS we described above. In this extreme, embryos would mistakenly be prioritized for implantation according to whether or not they share genetic variants with people exposed to higher levels of air pollution in a previous generation.

Similarly, popular methods to estimate genetic correlations (the correlation between two groups of individuals in genetic effects on a trait, or the correlation of genetic effects on two traits) often indiscriminately aggregate across genome-wide associations [95,96]. Such methods are useful for characterizing how the genetic bases of complex traits are intertwined. However, they may inadvertently mask (or even introduce) additional axes of confounding. For example, confounding by population stratification that is shared between the two groups or two traits, or confounding with genetic effects on a third trait, can lead to biases in genetic correlation estimates [67,77,84,97,98]. The use of genetic correlations in causal inference (e.g. in ‘Mendelian Randomization’ [99–101]) therefore remains controversial [77,102]. However, when a study reports conclusions based on *genetic correlations*, it is likely to be interpreted—particularly by non-experts—as unambiguously reflecting genetic causality.

## 4. Further confounding introduced via researchers’ choices

Such unknown axes of confounding are plausibly a concern in a recent study that, based on an analysis of genetic correlations, purported to resolve an evolutionary paradox: why alleles associated with same-sex sexual behaviour are maintained, despite being ‘reproductively disadvantageous’ [32]. In what follows, we focus on what we refer to as ‘analytical confounding’, where potential confounding is worsened, or even introduced, through researchers’ analysis choices. In [32]: confusing model assumptions with evidence, ignoring the compatibility of data with confounded explanations, and introducing confounding through researchers’ classification choices. We posit that, while these problems are not unique to genomic studies, they can evade attention when couched in reports about how behaviours and outcomes are *genetically correlated*.

### (a) Confusion of assumptions with evidence

**Figure 3.**
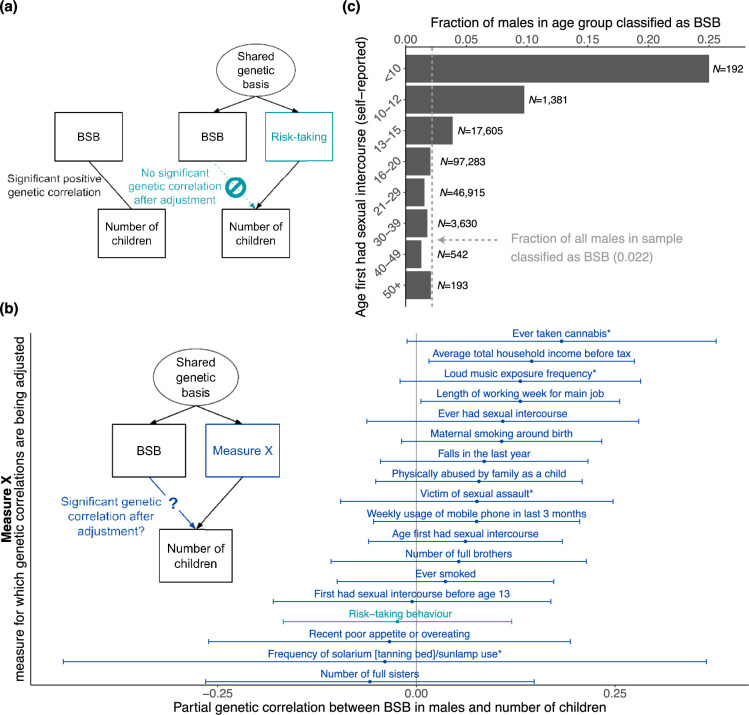
(a) Song & Zhang [32] show that the estimated genetic correlation between BSB (a measure of bisexual behaviour) in males and number of children is significantly different from zero (left diagram). They hypothesized the causal structure shown in the right diagram: genetic variants affecting BSB affect the number of children only through their simultaneous effect on risk-taking behaviour. When adjusting for genetic effects on risk-taking behaviour, the partial (residualized) genetic correlation between BSB and number of children is no longer significantly non-zero. They take this observation as evidence for their hypothesis. (b) When we repeat this analysis but replace risk taking with a variety of other measures (‘measure X,’ blue in causal diagram), 16 out of 18 measures we examined yield a partial genetic correlation between BSB and number of children that does not significantly differ from zero (bars show 95% confidence intervals). Therefore, the data are equally consistent with the hypothesis that genetic variants driving BSB are maintained through evolution as a byproduct of selection on any of these 16 measures. We note that most of these measures—like risk-taking behaviour—have a significant partial genetic correlation with number of children. Four of the measures, denoted with an asterisk, are not. (c) Male participants in the study sample who reported having first had sex before the age of 13 (including victims of childhood sexual assault, many of which would have had same-sex perpetrators) are likelier to be classified as BSB by the criteria used in Song & Zhang [32]. *N*, total number of males in each group.

In [32], a measure of bisexual behaviour is defined based on questionnaire data about total lifetime number of sexual partners and same-sex sexual partners (hereafter, we refer to this measure as BSB; see [103] and [104] for discussion of shortcomings of such measures). The paper reports a significant positive genetic correlation between BSB in males and the number of children. However, when adjusting this genetic correlation for genetic correlations of each measure with self-assessment as a ‘risk-taker’, the adjusted (or ‘partial’) genetic correlation between BSB in males and number of children was statistically indistinguishable from zero (figure 3a). It presents an interpretation of this finding as evidence that ‘the current genetic maintenance of male BSB is a byproduct of selection for male risk-taking behaviour.’ No explanation of the hypothesized mechanism by which risk-taking behaviour increases the number of offspring is given in [32], but in subsequent news coverage, one of the authors is quoted, ‘self-reported risk-taking [likely] includes unprotected sex and promiscuity, which could result in more children’ [105].

The study presents the contrast between these unadjusted and partial genetic correlations as support for a causal claim. However, the causal model is assumed *a priori*, and no evidence supporting this model is provided. Even under the assumption that the three measures considered are the only ones at play—and some causally affect others—the evidence is equally consistent with contradictory causal hypotheses (e.g. different directions of causality, arrows in fig. 2b of [32] and figure 3a; electronic supplementary material, figure S14 here; electronic supplementary material, note S8; c.f. [106]).

Furthermore, the study did not evaluate the support for any alternative model involving other factors, observed or latent. The authors justify their focus on risk-taking as a mechanistic explanation for the genetic maintenance of same-sex sexual behaviour by citing previous reports of genetic correlations of same-sex sexual behaviour and risk-taking [107,108]. However, these studies (and others [109]) reported multiple measures with similar (or even stronger) genetic correlations with same-sex sexual behaviour than risk-taking ([107]; electronic supplementary material, table S5). Sexual behaviour aside, [32] neither cites nor offers any evidence for the alleles associated with risk-taking being maintained over an evolutionary timescale. Additionally, this association is based on an answer to a single questionnaire question, ‘Would you describe yourself as someone who takes risks?’ [110, pg 5]. It is possible that responses to this question reflect a tendency towards practising unprotected sex and promiscuity, and that they simultaneously correlate with risk-taking tendencies that have been relevant for fitness throughout recent human evolution and across evolving societies; but, as acknowledged in [32], these key assumptions are hard to evaluate.

For these reasons, we asked: is there unique support for the assumed mechanistic model, in particular the role of risk-taking behaviour as a mediator? To answer this question, we considered models wherein a measure other than risk-taking mediates the genetic correlation between BSB and number of children (‘measure X’ in blue in figure 3b). If adjusting for this measure also results in a partial genetic correlation between BSB and number of children that is not significantly different from zero, the data are equally compatible with the hypothesis that there is a reproductive advantage for BSB-affecting alleles because of their simultaneous effect on measure X. We implemented this strategy with 18 measures, selected based on prior evidence of high genetic correlations with same-sex sexual behaviour, risk-taking behaviour and/or number of children ([109]; electronic supplementary material, tables S4, S5, note S8). All but two of these models yielded a partial genetic correlation between BSB and number of children that was not significantly different from zero (Genomic SEM [111] *p* > 0.05 before applying any correction for multiple testing; figure 3b). Hence, other causal narratives that do not involve risk taking could just as easily be constructed: the data are equally consistent with the hypothesis that genetic variants driving BSB are maintained through evolution as a byproduct of selection on the number of falls in the past year, usage of cell phone or any of these measures (figure 3b; electronic supplementary material, figure S15). The ease with which alternative hypotheses about genetic causality receive support using the method from [32] is revealing: the conclusion is highly reliant on the choice of a single hypothesis to test and a failure to consider confounding (box 1iv).

### (b) Confounding introduced by researchers’ classification choices

Measures relating to having experienced some form of nonconsensual sex (‘victim of sexual assault’ and ‘first had sex before age 13’) exhibited some of the strongest genetic correlations with BSB in males (electronic supplementary material, figure S15). This observation led us to be concerned about the ascertainment choices made in [32]. Indeed, we found that classification as a BSB individual is highly enriched among males who reported having first had sex before the age of 13 (in this regard, we note that children under this age cannot legally consent to any sexual activity in the UK [112]; figure 3c). Whereas 2.2% of males in the sample considered were classified as ‘BSB individuals,’ this classification rate was 9.8% among those who reported first having sex between the ages of 10−12 (inclusively), and 25% among those who reported first having had sex before the age of 10. For this dataset, there is no information about the age at which males classified as BSB first had same-sex sexual intercourse, or what fraction of victims of sexual assault had a perpetrator of the same sex. It is, however, established that the majority of reported sexual assaults on prepubescent male victims are carried out by male perpetrators [113–116]. This aggravates the concern that the BSB classification used in [32] conflated voluntary sexual behaviour and sexual assault, undermining the study’s stated aim of advancing our understanding of human sexual preferences. Taken together, our reanalysis of [32] cautions yet again against causal inference based on preferential attention towards sensational hypotheses and analyses that seemingly support them.

## 5. Conclusion

The study of the genetics underlying human behaviour and social outcomes, with its fraught history and heightened potential for misinterpretation and misappropriation [62,63,72,103,117,118], demands the utmost rigour. The failure to reckon with confounding fuels misinterpretation of genetics research and impedes scientific progress. We are therefore concerned that a publishing culture which rewards sensationalism may instead promote a decline in standards [119,120]. In that respect, everyone has a role to play: it is crucial that researchers, reviewers and editors uphold the highest standards in their handling of these complex, far-reaching issues.

## Data Availability

All code for reproducing our analyses is available on Zenodo [121]. Supplementary material is available online [122].

## References

[1] Galton F. 1869 Hereditary genius: an inquiry into its laws and consequences. New York, NY: MacMillan & Co. (10.1037/13474-000)

[2] Mehler B. 2015 Hereditarianism. In The Wiley Blackwell encyclopedia of race, ethnicity, and nationalism, pp. 1–3. Hoboken, NJ: John Wiley & Sons, Ltd. (10.1002/9781118663202)

[3] Clark G. 2023 The inheritance of social status: England, 1600 to 2022. Proc. Natl Acad. Sci. USA **120**, e2300926120. (10.1073/pnas.2300926120) 37364122 PMC10319028

[4] Wright S. 1931 Statistical methods in biology. J. Am. Stat. Assoc **26(173A)**, 155–163.

[5] Cavalli-Sforza L, Feldman MW. 1973 Models for cultural inheritance. I. Group mean and within group variation. Theor. Popul. Biol. **4**, 42–55. (10.1016/0040-5809(73)90005-1) 4726009

[6] Cavalli-Sforza LL, Feldman MW. 1973 Cultural versus biological inheritance: phenotypic transmission from parents to children. Am. J. Hum. Genet **25**, 618–637. 4797967 PMC1762580

[7] Rao DC, Morton NE, Yee S. 1974 Analysis of family resemblance. II. A linear model for familial correlation. Am. J. Hum. Genet. **26**, 331–359. 4857114 PMC1762612

[8] Rao DC, Morton NE, Yee S. 1976 Resolution of cultural and biological inheritance by path analysis. Am. J. Hum. Genet. **28**, 228–242. 944529 PMC1685018

[9] Cavalli-Sforza LL, Feldman MW. 1978 The evolution of continuous variation. III. Joint transmission of genotype, phenotype and environment. Genetics **90**, 391–425. (10.1093/genetics/90.2.391) 17248869 PMC1213897

[10] Rice J, Cloninger CR, Reich T. 1978 Multifactorial inheritance with cultural transmission and assortative mating I: description and basic properties of the unitary models. Am. J. Hum. Genet. **30**, 618–643. 747189 PMC1685878

[11] Cloninger CR, Rice J, Reich T. 1979 Multifactorial inheritance with cultural transmission and assortative mating II: a general model of combined polygenic and cultural inheritance. Am. J. Hum. Genet. **31**, 176–198. 453202 PMC1685756

[12] Cloninger CR, Rice J, Reich T. 1979 Multifactorial inheritance with cultural transmission and assortative mating III: family structure and the analysis of separation experiments. Am. J. Hum. Genet. **31**, 366–388. 572636 PMC1685778

[13] Rice J, Cloninger CR, Reich T. 1980 Analysis of behavioral traits in the presence of cultural transmission and assortative mating: applications to IQ and SES. Behav. Genet. **10**, 73–92. (10.1007/BF01067320) 7425997

[14] Lewontin RC, Rose S, Kamin LJ. 1984 Not in our genes: biology, ideology, and human nature. New York, NY: Pantheon Books.

[15] Vilhjálmsson BJ, Nordborg M. 2013 The nature of confounding in genome-wide association studies. Nat. Rev. Genet. **14**, 1–2. (10.1038/nrg3382) 23165185

[16] Feldman MW, Christiansen FB, Otto SP. 2013 Gene-culture co-evolution: teaching, learning, and correlations between relatives. Isr. J. Ecol. Evol. **59**, 72–91. (10.1080/15659801.2013.853435)

[17] Solon G. 2014 Theoretical models of inequality transmission across multiple generations. Res. Soc. Stratif. Mobil. **35**, 13–18. (10.1016/j.rssm.2013.09.005)

[18] Barton N, Hermisson J, Nordborg M. 2019 Why structure matters. eLife **8**, e45380. (10.7554/eLife.45380) 30895925 PMC6428565

[19] Uchiyama R, Spicer R, Muthukrishna M. 2022 Cultural evolution of genetic heritability. Behav. Brain Sci **45**, e152. (10.1017/S0140525X21000893) 34016199

[20] Collado MD, Ortuño-Ortín I, Stuhler J. 2023 Estimating intergenerational and assortative processes in extended family data. Rev. Econ. Stud. **90**, 1195–1227. (10.1093/restud/rdac060)

[21] Herzig AF, Noûs C, Pierre AS, Perdry H. 2023 A model for co-occurrent assortative mating and vertical cultural transmission and its impact on measures of genetic associations. bioRxiv (10.1101/2023.04.08.536101) 42297307

[22] Lewontin RC. 1974 Annotation: the analysis of variance and the analysis of causes. Am. J. Hum. Genet. **26**, 400–411. 4827368 PMC1762622

[23] Feldman MW, Lewontin RC. 1975 The heritability hang-up. Science **190**, 1163–1168. (10.1126/science.1198102) 1198102

[24] Bailey RC. 1997 Hereditarian scientific fallacies. Genetica **99**, 125–133. (10.1007/BF02259516) 9463068

[25] Holtzman NA. 2002 Genetics and social class. J. Epidemiol. Community Health **56**, 529–535. (10.1136/jech.56.7.529) 12080161 PMC1732191

[26] Feldman MW, Ramachandran S. 2018 Missing compared to what? Revisiting heritability, genes and culture. Phil. Trans. R. Soc. B **373**, 20170064. (10.1098/rstb.2017.0064) 29440529 PMC5812976

[27] Young AI, Benonisdottir S, Przeworski M, Kong A. 2019 Deconstructing the sources of genotype-phenotype associations in humans. Science **365**, 1396–1400. (10.1126/science.aax3710) 31604265 PMC6894903

[28] Shen H, Feldman MW. 2023 Drowning in shallow causality. Behav. Brain Sci. **46**, e199. (10.1017/S0140525X22002278) 37694932

[29] Goldberger AS. 1979 Heritability. Economica **46**, 327. (10.2307/2553675)

[30] Govindaraju DR, Goldstein AM. 2025 The elusive associations of nucleotides with human success: evolutionary genetics in education and social policies. Evo Edu Outreach **18(1)**, 4. (10.1186/s12052-025-00218-3)

[31] Lala KN, Feldman MW. 2024 Genes, culture, and scientific racism. Proc. Natl Acad. Sci. USA **121**, e2322874121. (10.1073/pnas.2322874121) 39556747 PMC11621800

[32] Song S, Zhang J. 2024 Genetic variants underlying human bisexual behavior are reproductively advantageous. Sci. Adv. **10**, eadj6958. (10.1126/sciadv.adj6958) 38170769 PMC10796114

[33] Greenland S, Pearl J, Robins JM. 1999 Causal diagrams for epidemiologic research. Epidemiology **10**, 37–48. 9888278

[34] Hernán MA, Robins JM. 2025 Causal inference: what if. Boca Raton, FL: CRC Press.

[35] Pearl J. 2009 Causality: models, reasoning, and inference. Cambridge, England: Cambridge University Press.

[36] VanderWeele TJ. 2013 Surrogate measures and consistent surrogates. Biometrics **69**, 561–565. (10.1111/biom.12071) 24073861 PMC4221255

[37] Pearl J. 1995 Causal diagrams for empirical research. Biometrika **82**, 702–710. (10.1093/biomet/82.4.702)

[38] Fisher RA. 1918 XV.—The correlation between relatives on the supposition of mendelian inheritance. Trans. R. Soc. Edinb. **52**, 399–433. (10.1017/s0080456800012163)

[39] Gimelfarb A. 1981 A general linear model for the genotypic covariance between relatives under assortative mating. J. Math. Biol. **13**, 209–226. (10.1007/bf00275215)

[40] Clark G. 2014 The son also rises. Princeton NJ: Princeton University Press.

[41] Clark G. 2023 As a hereditarian, I strongly support economic redistribution. Quillette. See https://quillette.com/2023/08/21/hereditarianism-and-economic-redistribution/.

[42] Odling-Smee FJ. 1988 Niche-constructing phenotypes. In The role of behavior in evolution, pp. 73–132. Cambridge, MA: The MIT Press.

[43] Hällsten M, Pfeffer FT. 2017 Grand advantage: family wealth and grandchildren’s educational achievement in Sweden. Am. Sociol. Rev. **82**, 328–360. (10.1177/0003122417695791) 29200464 PMC5703428

[44] Duncan GJ, Murnane RJ (eds). 2011 Whither opportunity?: rising inequality, schools, and children’s life chances. New York, NY: Russell Sage Foundation.

[45] Karagiannaki E. 2017 The effect of parental wealth on children’s outcomes in early adulthood. J. Econ. Inequal. **15**, 217–243. (10.1007/s10888-017-9350-1)

[46] Akee RKQ, Copeland WE, Keeler G, Angold A, Costello EJ. 2010 Parents’ incomes and children’s outcomes: a quasi-experiment using transfer payments from casino profits. Am. Econ. J. **2**, 86–115. (10.1257/app.2.1.86) PMC2891175 20582231

[47] Cooper K, Stewart K. 2021 Does household income affect children’s outcomes? A systematic review of the evidence. Child Indic. Res. **14**, 981–1005. (10.1007/s12187-020-09782-0)

[48] Cavalli-Sforza LL, Feldman MW. 1981 Cultural transmission and evolution: a quantitative approach. Princeton, NJ: Princeton University Press. 7300842

[49] Turkheimer E. 2000 Three laws of behavior genetics and what they mean. Curr. Dir. Psychol. Sci. **9**, 160–164. (10.1111/1467-8721.00084)

[50] Young AI *et al*. 2018 Relatedness disequilibrium regression estimates heritability without environmental bias. Nat. Genet. **50**, 1304–1310. (10.1038/s41588-018-0178-9) 30104764 PMC6130754

[51] Yengo L *et al*. 2018 Imprint of assortative mating on the human genome. Nat. Hum. Behav. **2**, 948–954. (10.1038/s41562-018-0476-3) 30988446 PMC6705135

[52] Causa O, Johansson Å. 2009 Intergenerational Social Mobility. OECD Economics Department Working Papers (10.1787/223106258208)

[53] Chetty R, Hendren N, Kline P, Saez E. 2014 Where is the land of opportunity? The geography of intergenerational mobility in the United States. Q. J. Econ. **129**, 1553–1623. (10.1093/qje/qju022)

[54] Miles A. 1999 Social mobility in nineteenth- and early twentieth-century England. London, UK: Palgrave Macmillan. (10.1057/9780230373211)

[55] Lee JJ. 2023 The heritability and persistence of social class in England. Proc. Natl Acad. Sci. USA **120**, e2309250120. (10.1073/pnas.2309250120) 37406089 PMC10629509

[56] Cosh C. 2023 Is socioeconomic status hereditary?. National Post. See https://nationalpost.com/opinion/is-socioeconomic-status-hereditary.

[57] Marks GN. 2023 Has cognitive ability become more important for education and the labor market? A comparison of the project talent and 1979 national longitudinal survey of youth cohorts. J. Intell. **11**, 169. (10.3390/jintelligence11080169) 37623552 PMC10455275

[58] Ratia J. 2024 El Estatus Social También se Hereda. Ethic. See https://ethic.es/el-estatus-social-tambien-se-hereda.

[59] Mayo O, Nanjundiah V. 2024 Reflections on assortative mating, social stratification, and genetics. J. Genet **103(1)**, 15. (10.1007/s12041-024-01467-9) 38644559

[60] Scarr S, McCartney K. 1983 How people make their own environments: a theory of genotype greater than environment effects. Child Dev. **54**, 424–435. (10.1111/j.1467-8624.1983.tb03884.x) 6683622

[61] Feldman M. 2014 Echoes of the past: hereditarianism and a troublesome inheritance. PLoS Genet. **10**, e1004817. (10.1371/journal.pgen.1004817) 25502763 PMC4263368

[62] Coop G, Przeworski M. 2022 Lottery, luck, or legacy. A review of ‘The Genetic Lottery: Why DNA matters for social equality’. Evolution **76**, 846–853. (10.1111/evo.14449)

[63] Coop G, Przeworski M. 2022 Luck, lottery, or legacy? The problem of confounding. A reply to Harden. Evolution **76**, 2464–2468. (10.1111/evo.14588) 35915930 PMC9627830

[64] Young AS. 2023 Estimation of indirect genetic effects and heritability under assortative mating. BioRxiv (10.1101/2023.07.10.548458)

[65] Berg JJ *et al*. 2019 Reduced signal for polygenic adaptation of height in UK Biobank. Elife **8**, e39725. (10.7554/elife.39725) 30895923 PMC6428572

[66] Sohail M *et al*. 2019 Polygenic adaptation on height is overestimated due to uncorrected stratification in genome-wide association studies. Elife **8**, e39702. (10.7554/elife.39702) 30895926 PMC6428571

[67] Mostafavi H, Harpak A, Agarwal I, Conley D, Pritchard JK, Przeworski M. 2020 Variable prediction accuracy of polygenic scores within an ancestry group. Elife **9**, e48376. (10.7554/elife.48376) 31999256 PMC7067566

[68] Lander ES, Schork NJ. 1994 Genetic dissection of complex traits. Science **265**, 2037–2048. (10.1126/science.8091226) 8091226

[69] Veller C, Coop GM. 2024 Interpreting population- and family-based genome-wide association studies in the presence of confounding. PLoS Biol. **22**, e3002511. (10.1371/journal.pbio.3002511) 38603516 PMC11008796

[70] Nishimura KK *et al*. 2013 Early-life air pollution and asthma risk in minority children. The GALA II and SAGE II studies. Am. J. Respir. Crit. Care Med. **188**, 309–318. (10.1164/rccm.201302-0264oc) 23750510 PMC3778732

[71] Okbay A *et al*. 2022 Polygenic prediction of educational attainment within and between families from genome-wide association analyses in 3 million individuals. Nat. Genet. **54**, 437–449. (10.1038/s41588-022-01016-z) 35361970 PMC9005349

[72] Meyer MN *et al*. 2023 Wrestling with social and behavioral genomics: risks, potential benefits, and ethical responsibility. Hastings Cent. Rep. **53**, S2–S49. (10.1002/hast.1477) PMC10433733 37078667

[73] Nivard MG, Belsky DW, Harden KP, Baier T, Andreassen OA, Ystrøm E, van Bergen E, Lyngstad TH. 2024 More than nature and nurture, indirect genetic effects on children’s academic achievement are consequences of dynastic social processes. Nat. Hum. Behav. **8**, 771–778. (10.1038/s41562-023-01796-2) 38225408 PMC11569812

[74] Sella G, Barton NH. 2019 Thinking about the evolution of complex traits in the era of genome-wide association studies. Annu. Rev. Genomics Hum. Genet. **20**, 461–493. (10.1146/annurev-genom-083115-022316) 31283361

[75] Zaidi AA, Mathieson I. 2020 Demographic history mediates the effect of stratification on polygenic scores. Elife **9**, e61548. (10.7554/elife.61548) 33200985 PMC7758063

[76] Wang H, Aragam B, Xing EP. 2022 Trade-offs of linear mixed models in genome-wide association studies. J. Comput. Biol. **29**, 233–242. (10.1089/cmb.2021.0157) 35230156 PMC8968846

[77] Border R *et al*. 2022 Cross-trait assortative mating is widespread and inflates genetic correlation estimates. Science **378**, 754–761. (10.1126/science.abo2059) 36395242 PMC9901291

[78] Young AI *et al*. 2022 Mendelian imputation of parental genotypes improves estimates of direct genetic effects. Nat. Genet. **54**, 897–905. (10.1038/s41588-022-01085-0) 35681053 PMC9197765

[79] Onifade M, Roy-Gagnon MH, Parent MÉ, Burkett KM. 2022 Comparison of mixed model based approaches for correcting for population substructure with application to extreme phenotype sampling. BMC Genom. **23**, 98. (10.1186/s12864-022-08297-y) PMC8815214 35120458

[80] Yao Y, Ochoa A. 2023 Limitations of principal components in quantitative genetic association models for human studies. eLife **12**, e79238. (10.7554/eLife.79238) 37140344 PMC10234632

[81] Aw AJ, McRae J, Rahmani E, Song YS. 2024 Highly parameterized polygenic scores tend to overfit to population stratification via random effects. BioRxiv (10.1101/2024.01.27.577589)

[82] Grinde KE, Browning BL, Reiner AP, Thornton TA, Browning SR. 2024 Adjusting for principal components can induce spurious associations in genome-wide association studies in admixed populations. BioRxiv (10.1101/2024.04.02.587682) PMC11684764 39680601

[83] Smith SP, Smith OS, Mostafavi H, Peng D, Berg JJ, Edge MD, Harpak A. 2025 A litmus test for confounding in polygenic scores. BioRxiv (10.1101/2025.02.01.635985)

[84] Zabad S, Ragsdale AP, Sun R, Li Y, Gravel S. 2021 Assumptions about frequency‐dependent architectures of complex traits bias measures of functional enrichment. Genet. Epidemiol. **45**, 621–632. (10.1002/gepi.22388) 34157784

[85] LaPierre N, Fu B, Turnbull S, Eskin E, Sankararaman S. 2023 Leveraging family data to design Mendelian randomization that is provably robust to population stratification. Genome Res. **33**, 1032–1041. (10.1101/gr.277664.123) 37197991 PMC10538495

[86] Giangrande EJ, Turkheimer E. 2022 Race, ethnicity, and the Scarr-Rowe hypothesis: a cautionary example of fringe science entering the mainstream. Perspect. Psychol. Sci. **17**, 696–710. (10.1177/17456916211017498) 34793248

[87] Turkheimer E, Rodock Greer S. 2024 Spit for science and the limits of applied psychiatric genetics. Philos. Psychiatr. Psychol **31(4)**, 397–424. (10.1353/ppp.0.a923702)

[88] Spielman RS, McGinnis RE, Ewens WJ. 1993 Transmission test for linkage disequilibrium: the insulin gene region and insulin-dependent diabetes mellitus (IDDM). Am. J. Hum. Genet. **52**, 506–516. 8447318 PMC1682161

[89] Lee JJ *et al*. 2018 Gene discovery and polygenic prediction from a genome-wide association study of educational attainment in 1.1 million individuals. Nat. Genet. **50**, 1112–1121. (10.1038/s41588-018-0147-3) 30038396 PMC6393768

[90] Trejo S, Domingue BW. 2018 Genetic nature or genetic nurture? Introducing social genetic parameters to quantify bias in polygenic score analyses. Biodemography Soc. Biol. **64**, 187–215. (10.1080/19485565.2019.1681257) 31852332

[91] Selzam S, Ritchie SJ, Pingault JB, Reynolds CA, O’Reilly PF, Plomin R. 2019 Comparing within- and between-family polygenic score prediction. Am. J. Hum. Genet. **105**, 351–363. (10.1016/j.ajhg.2019.06.006) 31303263 PMC6698881

[92] Howe LJ *et al*. 2022 Within-sibship genome-wide association analyses decrease bias in estimates of direct genetic effects. Nat. Genet. **54**, 581–592. (10.1038/s41588-022-01062-7) 35534559 PMC9110300

[93] Torkamani A, Wineinger NE, Topol EJ. 2018 The personal and clinical utility of polygenic risk scores. Nat. Rev. Genet. **19**, 581–590. (10.1038/s41576-018-0018-x) 29789686

[94] Vilhjálmsson BJ *et al*. 2015 Modeling linkage disequilibrium increases accuracy of polygenic risk scores. Am. J. Hum. Genet. **97**, 576–592. (10.1016/j.ajhg.2015.09.001) 26430803 PMC4596916

[95] Yang J *et al*. 2010 Common SNPs explain a large proportion of the heritability for human height. Nat. Genet. **42**, 565–569. (10.1038/ng.608) 20562875 PMC3232052

[96] Bulik-Sullivan B *et al*. 2015 An atlas of genetic correlations across human diseases and traits. Nat. Genet. **47**, 1236–1241. (10.1038/ng.3406) 26414676 PMC4797329

[97] van Rheenen W, Peyrot WJ, Schork AJ, Lee SH, Wray NR. 2019 Genetic correlations of polygenic disease traits: from theory to practice. Nat. Rev. Genet. **20**, 567–581. (10.1038/s41576-019-0137-z) 31171865

[98] Morris TT, Davies NM, Hemani G, Smith GD. 2020 Population phenomena inflate genetic associations of complex social traits. Sci. Adv. **6**, eaay0328. (10.1126/sciadv.aay0328) 32426451 PMC7159920

[99] Davey Smith G, Hemani G. 2014 Mendelian randomization: genetic anchors for causal inference in epidemiological studies. Hum. Mol. Genet. **23**, R89–R98. (10.1093/hmg/ddu328) 25064373 PMC4170722

[100] Sanderson E *et al*. 2022 Mendelian randomization. Nat. Rev. Methods Prim **2(1)**, 6. (10.1038/s43586-021-00092-5) PMC7614635 37325194

[101] Richmond RC, Davey Smith G. 2022 Mendelian randomization: concepts and scope. Cold Spring Harb. Perspect. Med. **12**, a040501. (10.1101/cshperspect.a040501) 34426474 PMC8725623

[102] Hu X, Cai M, Xiao J, Wan X, Wang Z, Zhao H, Yang C. 2024 Benchmarking Mendelian randomization methods for causal inference using genome-wide association study summary statistics. Am. J. Hum. Genet. **111**, 1717–1735. (10.1016/j.ajhg.2024.06.016) 39059387 PMC11339627

[103] Ventresca C, Martschenko DO, Wedow R, Civelek M, Tabery J, Carlson J, Parker SCJ, Ramos PS. 2024 The methodological and ethical concerns of genetic studies of same-sex sexual behavior. Am. J. Hum. Genet. **111**, 2107–2116. (10.1016/j.ajhg.2024.08.007) 39255798 PMC11480801

[104] Vázquez IG. 2022 The gay gene(s)? Rethinking the concept of sexual orientation in the context of science. Biol. Philos **37(5)**, 45. (10.1007/s10539-022-09875-w)

[105] dela Cruz Tan MD. 2024 Genetic variants underlying male bisexual behavior, risk-taking linked to more children – study. OutrageMag. See https://outragemag.com/genetic-variants-underlying-male-bisexual-behavior-risk-taking-linked-to-more-children-study/.

[106] Bullock JG, Green DP, Ha SE. 2010 Yes, but what’s the mechanism? (don’t expect an easy answer). J. Pers. Soc. Psychol. **98**, 550–558. (10.1037/a0018933) 20307128

[107] Ganna A *et al*. 2019 Large-scale GWAS reveals insights into the genetic architecture of same-sex sexual behavior. Science **365**, eaat7693. (10.1126/science.aat7693) 31467194 PMC7082777

[108] Zietsch BP *et al*. 2021 Genomic evidence consistent with antagonistic pleiotropy may help explain the evolutionary maintenance of same-sex sexual behaviour in humans. Nat. Hum. Behav. **5**, 1251–1258. (10.1038/s41562-021-01168-8) 34426668

[109] Neale Lab. 2018 UKBB genetic correlation browser. UK Biobank Genetic Correlation Browser. See https://ukbb-rg.hail.is/.

[110] Bycroft C *et al*. 2018 The UK Biobank resource with deep phenotyping and genomic data. Nature **562**, 203–209. (10.1038/s41586-018-0579-z) 30305743 PMC6786975

[111] Grotzinger AD *et al*. 2019 Genomic structural equation modelling provides insights into the multivariate genetic architecture of complex traits. Nat. Hum. Behav. **3**, 513–525. (10.1038/s41562-019-0566-x) 30962613 PMC6520146

[112] UK Government. 2003 Sexual Offences Act. See https://www.legislation.gov.uk/ukpga/2003/42/contents.

[113] Gil S. 2014 Male victims of childhood sexual abuse by a male or female perpetrator. J. Trauma. Stress Disord. Treat **3(03)**, 2. (10.4172/2324-8947.1000128)

[114] Mohler-Kuo M, Landolt MA, Maier T, Meidert U, Schönbucher V, Schnyder U. 2014 Child sexual abuse revisited: a population-based cross-sectional study among Swiss adolescents. J. Adolesc. Health **54**, 304–311.(10.1016/j.jadohealth.2013.08.020) 24182941

[115] Ferragut M, Ortiz-Tallo M, Blanca MJ. 2021 Victims and perpetrators of child sexual abuse: abusive contact and penetration experiences. Int. J. Environ. Res. Public Health **18**, 9593. (10.3390/ijerph18189593) 34574520 PMC8472568

[116] Mathews B *et al*. 2024 Child sexual abuse by different classes and types of perpetrator: prevalence and trends from an Australian national survey. Child Abus. Negl. **147**, 106562. (10.1016/j.chiabu.2023.106562) 38061281

[117] Panofsky A. 2014 Misbehaving science: controversy and the development of behavior genetics. Chicago, IL: University of Chicago Press.

[118] Carlson J, Henn BM, Al-Hindi DR, Ramachandran S. 2022 Counter the weaponization of genetics research by extremists. Nature **610**, 444–447. (10.1038/d41586-022-03252-z) 36261568

[119] Caulfield T. 2018 Spinning the genome: why science hype matters. Perspect. Biol. Med. **61**, 560–571. (10.1353/pbm.2018.0065) 30613038

[120] Hopf H, Matlin SA, Mehta G, Krief A. 2020 Blocking the hype‐hypocrisy‐falsification‐fakery pathway is needed to safeguard science. Angew. Chem. **132**, 2170–2174. (10.1002/ange.201911889) 31589804

[121] Benning J, Carlson J. 2025 harpak-lab/confounding: v1.1. Zenodo. (10.5281/zenodo.16994853)

[122] Benning JW, Carlson J, Smith OS, Shaw RG, Harpak A. 2025 Supplementary material from: Confounding fuels misinterpretation in human genetics. Figshare. (10.6084/m9.figshare.c.8082266) PMC12587318 41190516

